# *Rhodobacter azotoformans* LPS (RAP99-LPS) Is a TLR4 Agonist That Inhibits Lung Metastasis and Enhances TLR3-Mediated Chemokine Expression

**DOI:** 10.3389/fimmu.2021.675909

**Published:** 2021-05-25

**Authors:** Kaoru Murakami, Daisuke Kamimura, Rie Hasebe, Mona Uchida, Nobuya Abe, Reiji Yamamoto, Jing-Jing Jiang, Yasuhiro Hidaka, Yuko Nakanishi, Shuzo Fujita, Yuki Toda, Nobuhiro Toda, Hiroki Tanaka, Shizuo Akira, Yuki Tanaka, Masaaki Murakami

**Affiliations:** ^1^ Division of Molecular Psychoimmunology, Institute for Genetic Medicine, Graduate School of Medicine, Hokkaido University, Sapporo, Japan; ^2^ Institute of Preventive Genomic Medicine, School of Life Sciences, Northwest University, Xian, China; ^3^ TFK Co., Ltd, Kobe, Japan; ^4^ Laboratory of Host Defense, World Premier Institute Immunology Frontier Research Center, Osaka University, Osaka, Japan

**Keywords:** RAP99-LPS, TLR4, agonist, NF-kB, STAT3

## Abstract

The lipopolysaccharides (LPSs) of *Rhodobacter* are reported to be TLR4 antagonists. Accordingly, the extract of *Rhodobacter azotoformans* (RAP99) is used as a health supplement for humans and animals in Japan to regulate immune responses *in vivo*. We previously analyzed the LPS structure of RAP99 (RAP99-LPS) and found it is different from that of *E. coli*-LPS but similar to lipid A from *Rhodobacter sphaeroides* (RSLA), a known antagonist of TLR4, with both having three C14 fatty acyl groups, two C10 fatty acyl groups, and two phosphates. Here we show that RAP99-LPS has an immune stimulatory activity and acts as a TLR4 agonist. Pretreatment of RAP99-LPS suppressed *E. coli*-LPS-mediated weight loss, suggesting it is an antagonist against *E. coli*-LPS like other LPS isolated from *Rhodobacter*. However, injections of RAP99-LPS caused splenomegaly and increased immune cell numbers in C57BL/6 mice but not in C3H/HeJ mice, suggesting that RAP99-LPS stimulates immune cells *via* TLR4. Consistently, RAP99-LPS suppressed the lung metastasis of B16F1 tumor cells and enhanced the expression of TLR3-mediated chemokines. These results suggest that RAP99-LPS is a TLR4 agonist that enhances the activation status of the immune system to promote anti-viral and anti-tumor activity *in vivo*.

## Introduction

Lipopolysaccharides (LPSs) are a component of Gram-negative bacteria cell walls. *E. coli*-derived LPS (*E. coli*-LPS) is known to stimulate immune cells, especially innate immune cells including dendritic cells (DCs) and macrophages, *via* Toll-like receptor (TLR)4 molecules (1). Members of the TLR family recognize conserved microbial structures and activate not only innate immune responses but also adaptive immune responses through DC-mediated T cell activation (2). After TLR4 binds LPS, at least two signaling pathways are activated. One is dependent on myeloid differentiation factor 88 (Myd88) and the other on TIR domain-containing adapter-inducing IFNb (TRIF) (3). Myd88, a TLR-binding protein, contributes to NF-kB activation, which induces inflammatory cytokines such as IL-6, TNFa, and IL-1b. On the other hand, TRIF-dependent signaling leads to IFNg expression *via* the activation of IRF3 (4). TLR3 plays a role in recognizing the double-stranded RNA of infecting viruses, including single-stranded viruses, such as coronavirus, when the viruses form double-stranded RNA ephemerally during replication (5, 6). TLR4 signaling induces TLR3 upregulation in macrophages (7), and we found a similar effect on endothelial cells (unpublished results). Moreover, TLR4 is internalized by binding to LPS, and LPS-bound TLR4 and TLR3 are both localized at the endosome. The two TLRs also share a common signaling pathway involving TRIF (8). TRIF, which is a binding partner for TLR3 and TLR4, contributes to innate immunity by binding to TBK1 and TRAF3, which activates IRF3 and IRF7 to induce type-I interferon production, and by interacting with TRAF6 to activate NF-kB (9).

*Rhodobacter* sp. are also Gram-negative bacteria and reported to produce LPSs that lack endotoxic activity and act as antagonists of other LPSs including *E. coli*-LPS (10–12). Additionally, they induce low levels of inflammatory cytokines such as IL-6. The structure of *Rhodobacter azotoformans* LPS, RAP99-LPS, contains a novel lipooligosaccharide consisting of a short glycan structure containing glucuronic acid (13). However, no functional analysis of RAP99-LPS has been performed. In this study, we investigated the TLR4 antagonist functions of RAP99-LPS. We showed that RAP99-LPS activated various immune cells including DC and T cells *via* TLR4, while its pretreatment suppressed *E. coli*-LPS-induced weight loss *in vivo* in mice. Furthermore, RAP99-LPS inhibited the metastasis of B16F1 cancer cells in the lung. RAP99-LPS pretreatment suppressed the TLR3-mediated expression of NF-kB and STAT3 stimulators, including IL-1b, TNFa, IL-17A, and IL-6, but enhanced the expression of chemokines such as MCP1, MIP1a, and MIP1b. These findings indicate that RAP99-LPS is in fact a TLR4 stimulator and not an antagonist, having beneficial effects *in vivo*, including weaker TLR4-mediated endotoxin shock and TLR3-induced cytokine storm and suppressed lung tumor metastasis.

## Materials and Methods

### Mouse Strains

C57BL/6 mice, C3H/HeN and C3H/HeJ male mice (6–9 weeks) were purchased from Japan SLC (Shizuoka, Japan). All animal experiments performed in this study were approved by the Institute for Genetic Medicine, Hokkaido University, Japan, and all mice were housed and maintained under specific pathogen-free conditions according to the institute’s animal care guidelines. Animal experiments were performed following the guidelines of the Institutional Animal Care and Use Committees of Hokkaido University. The protocols for animal experiments were approved by the Institutional Animal Care and Use Committees of Hokkaido University.

### Regents

RAP99-LPS was isolated as described previously (13). In brief, the strains of *R. azotoformans* BP0899 used in this study were deposited in the Nite Biological Resource Center (accession number: NITE BP-644). RNase A, DNase, and proteinase K were purchased from Merck (Darmstadt, Germany). Bacto Yeast Extract was purchased from BD Difco (New Jersey, USA). SA medium was composed of sodium succinate (1.0 g), sodium acetate (1.0 g), KH_2_PO_4_ (0.5 g), K_2_HPO_4_ (0.6 g), (NH_4_)_2_SO_4_ (1.0 g), MgSO_4_ 7H_2_O (0.2 g), NaCl (0.2 g), CaCl_2_ 2H_2_O (0.05 g) Bacto Yeast Extract (0.1 g), trace element solution (1 ml), and vitamin solution (1 ml) in 1,000 ml of distilled water. The trace element solution was composed of EDTA-2Na (1,000 mg), FeCl_3_ 6H_2_O (2,000 mg), ZnCl_2_ (100 mg), MnCl_2_ 4H_2_O (100 mg), H_3_BO_3_ (100 mg), CoCl_2_ 6H2O (100 mg), Na_2_MoO_4_ 2H_2_O (20 mg), CuCl_2_ 2H_2_O (10 mg), NiCl_2_ 6H2O (10 mg), and Na_2_SeO_3_ (5 mg) in 1,000 ml of distilled water. The vitamin solution was composed of thiamine-HCl (50 mg), niacin (50 mg), p-aminobenzoic acid (30 mg), pyridoxine-HCl (10 mg), biotin (5 mg), and vitamin B_12_ (5 mg) in 100 ml of distilled water. The vitamin solution was stocked at 5°C in the dark. SA medium (pH 6.7) was autoclaved at 121°C for 15 min. Solvents used in the mass spectrometry analysis were HPLC grade or LC/MS grade. Other solvents and reagents were special grade. TLC plates (silica gel, 60 F254) were purchased from Merck Millipore (Darmstadt, Germany).

*R. azotoformans* was grown aerobically in SA medium at 30°C. The culture was harvested by centrifugation and lyophilized. Lyophilized bacterial cells (20.0 g) were washed with acetone until the brownish color disappeared. The cells were then dried *in vacuo* and subjected to the hot phenol–water method. The extract was centrifuged at 27,200×*g* at 4°C for 40 min three times. The supernatant was separated and dialyzed (MWCO 7 kDa) using distilled water until no phenol was detected. The obtained extract was concentrated to 75 ml by ultrafiltration (MWCO 100 kDa). RNase A (0.5 mg/ml) and DNase (5 μg/ml) were added to the extract, and the resulting solution was incubated at 37°C for 6 h. Next, 200 μg/ml of proteinase K was added, and the solution was incubated at 50°C for 4 h. After the degradation of nucleic acids and proteins, the aqueous layer was centrifuged at 1,720×*g* to obtain a precipitate. 30 ml of distilled water was then added to the remaining precipitate, which was dissolved by stirring. This aqueous solution was subjected to ultra-filtration (MWCO 100 kDa). Afterwards, the aqueous solution in which the precipitate fraction was dissolved was subjected to the hot phenol–water method three times followed by dialysis (MWCO 7 kDa) and ultrafiltration (MWCO 100 kDa). The resultant was lyophilized to give 165 mg of purified LOS.

*E. coli*-LPS (055: B5) (2% protein and 2.5% nucleic acid contamination) was purchased from Merck (Darmstadt, Germany).

### Cell Lines and TLR2 and TLR4 Signal Assays

A type 1 collagen+ endothelial BC1 cell line was obtained from Dr. M. Miyasaka (Osaka University, Suita, Japan) (14). BC1 cells were originally established as follows: Femurs of BALB/c mice (Charles River Japan Inc., Kanagawa, Japan) were isolated and washed with phosphate-buffered saline (PBS). After removing the smooth muscle and periosteum, bone marrow cells were collected. The remaining bone was cut into pieces and treated with 0.5% collagenase in Hanks’ balanced salt solution without calcium and magnesium for 15 min at 37°C with gentle shaking. The removed cells (BC1 cells) were collected and cultured in 10% FBS high-glucose DMEM (15).

HEK-Blue™ mTLR2 and HEK-Blue™ mTLR4 cells were purchased from Invivogen (San Diego, America). The cells were maintained and cultured in growth medium supplemented with 1× HEK-Blue selection, and the growth medium was renewed once a week. After 70–80% confluency was reached, the growth medium was discarded, and the cells were rinsed with pre-warmed PBS. Then, the cells were counted, a cell suspension with 280,000 cells/ml in HEK-Blue Detection medium was prepared, and 180 μl of the cell suspension and 20 μl of the sample or negative control or positive control to each well of a 96-well plate was immediately added to each well of 96-well plate. The plate was incubated at 37°C in 5% CO_2_ for 9 h. Finally, each well was analyzed using an iMark Microplate reader spectrophotometer (Bio-Rad, Tokyo, Japan) at 630 nm.

### LPS-Mediated Endotoxin Model

C57BL/6 mice, C3H/HeN and C3H/HeJ mice were intraperitoneally injected with 200 μg of RAP99-LPS and/or *E. coli*-LPS followed by body weight measurements.

### Tumor Metastasis Model

The experimental metastasis assay was performed as previously described using B16F1 cells (16). In brief, we maintained and cultured B16F1 cells in RPMI 1640 with 10% FBS in a 10-cm Petri dish. After 70–80% confluency was reached, B16F1 cells were harvested, washed three times with serum-free RPMI 1640, and resuspended at 1 × 10^7^ cells/ml in saline. B16F1 cells (1 × 10^6^/100 μl) were injected i.v. into C57BL6 mice. Then, RAP99-LPS (0.3 mg/300 μl) or saline (300 μl) was intraperitoneally injected every 24 h. For the RAP99-LPS group, drinking water was replaced with water in which RAP99-LPS was dissolved (10 × 10^−2^ mg/ml). On day 14, the number of tumor colonies in the lungs was counted under a dissecting microscope (Olympus, Tokyo, Japan).

### Flow Cytometry and Cell Preparation

Spleens were collected from mice treated with *E. coli*-LPS, RAP99-LPS, or saline. The resulting spleens were crushed on a cell strainer in a 50 ml tube with DMEM media. The obtained splenocytes were centrifuged, the supernatant was discarded, and 1 ml of NH_4_Cl for 1 min on ice was added to the precipitate. For the Ter-119 analysis, the NH_4_Cl treatment was omitted. 49 ml DMEM was then added, and the mixture was centrifuged. The cell pellet was resuspended with MACS Buffer to obtain a single-cell suspension. Cell numbers in the single-cell suspension were measured with a blood cell meter followed by flow cytometry analysis. Cell surface antigens of the splenocyte populations were stained in the presence of 2.4G2 antibody using the following anti-mouse antibodies: FITC-conjugated anti-CD19 (clone MB19-1, eBioscience, Tokyo, Japan), Pacific Blue-conjugated anti-CD3 (clone 145-2C11; BioLegend, Tokyo, Japan), PE-Cy7-conjugated anti-CD4 (clone RM4-5; eBioscience), FITC-conjugated anti-CD44 (clone IM7; eBioscience), APC-conjugated anti-CD11c (clone N418; BioLegend), APC-conjugated anti-MHC class II (clone M5/114.15.2; BioLegend), FITC-conjugated anti-CD11b (clone M1/70, BioLegend), Pacific Blue-conjugated anti-MHC class II (clone AF6-120.1; BioLegend), PE-conjugated anti-CD45R/B220 (clone RA3-6B2; BD Biosciences Pharmingen, San Diego, CA, USA), Pacific Blue-conjugated anti-Gr-1 (clone RB6-8C5, BioLegend), biotin-conjugated anti-CD11b (clone M1/70, BioLegend), anti-CD19 (clone 6D5, eBioscience), and anti-NK1.1 (clone PK136, eBioscience). The gating strategies are shown in **Supplementary Figures 1**–**4**.

Regarding intracellular Foxp3 staining, a single-cell suspension of 1–5 × 10^5^cells/ml was prepared and stained with surface markers, including CD4, CD25, and MHC class II as a negative marker, in a 96-well V bottom plate. After washing with MACS Buffer (5% FBS-PBS with 1.86g/l EDTA), cell pellets were added with 200 µl of Foxp3 Fixation/Permeabilization working solution to each well. The cell suspensions were incubated for 30–60 min at 2–8°C in the dark. The resulting samples were centrifuged at 400–600×*g* for 5 min, the supernatant was discarded, and the precipitate was vortexed. The cells were washed with 200 µl 1× Permeabilization Buffer twice. About 100 µl of 1× Permeabilization Buffer was added with 0.5 µl of fluorescence conjugated anti-Foxp3 antibody (clone MF23, Becton, Dickinson and Company, New Jersey, America), and the mixture was incubated for 60 min at room temperature. The cells were then washed with 200 µl of 1× Permeabilization Buffer twice. The stained cells were resuspended in MACS Buffer followed by flow cytometry analysis.

### Bioplex Assay of Cytokines

Bioplex assays were performed as previously described (17). In brief, antibody-conjugated beads were put into the wells of a 96-well plate and mixed with 50 μl of serum samples and standards. The plate was then shaken at 850 rpm for 30 min at room temperature. The samples were washed three times, 25 μl of detection antibody was added, and the plate was shaken at 850 rpm at room temperature for 30 min. The samples were washed again three times, 50 μl streptavidin-PE was added to each well, and the plate was shaken again at 850 rpm at room temperature for 10 min. Then, 125 μl assay buffer was added, and the plate was shaken at 850 rpm at room temperature for 30 min. Finally, the plate was read using a bio-plex analyzer (Bio-Rad, Tokyo).

### Immunohistochemistry

To confirm the activation of the IL-6 amplifier, we performed immunohistochemistry of anti-phospho-STAT3 (#9138, Cell Signaling Technology, Massachusetts, America) and phospho-NF-kB antibodies (SAB4504488, Merck) using paraffin sections of the spleen after the stimulations. Image quantification was performed in four areas/sections in each mouse. Representative data are shown (n > 3). The area ratio of nuclei with pStat3 or pp65 (DAB + area) is presented per 10,000µm^2^ (Hematoxylin+ area).

### Statistical Analysis

Student’s t tests (two-tailed) were used for the statistical analysis of differences between two groups. Two-way ANOVA with Dunnett’s *post hoc* analysis was used for multiple comparisons. *P* values less than 0.05 were considered significant (*P < 0.05, **P < 0.01, and ***P < 0.001).

## Results

### RAP99-LPS Is a TLR4 Ligand With Antagonist Activity to *E. coli*-LPS

**Figure 1:**
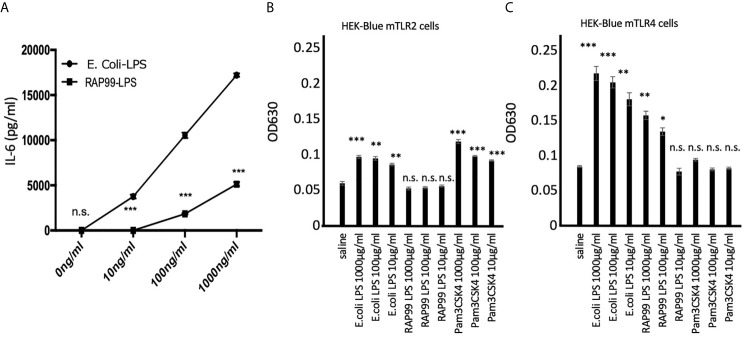
RAP99-LPS stimulates TLR4 but not TLR2 *in vitro*. **(A)** IL-6 concentration in the culture supernatant by ELISA. BC1 cells were treated with *E.coli*-LPS or RAP99-LPS for 24 h prior to the analysis. **(B)** OD630 was analyzed 6 h after treating HEK-Blue™ mTLR2 and **(C)** HEK-Blue™ mTLR4 cells with *E. coli*-LPS or RAP99-LPS. Mean scores ± SD are shown. *p < 0.05, **p < 0.01, ***p < 0.005; n.s., not significant; Student’s t tests. (Representative data are shown.)

We purified RAP99-LPS using the standard method for *E. coli*-LPS purification (13). The concentrations of protein and nucleic acid were similar to Sigma *E. coli*-LPS (2.1 and 2.5%, respectively), indicating good purity. We then investigated the *in vitro* responses of the two LPS. We found that RAP99-LPS increased IL-6 concentration dose dependently but not as strongly as *E. coli*-LPS (**Figure 1A**). Because the most abundant contaminating TLR activators in purified LPS are TLR2 ligands (18), we investigated whether the RAP99-LPS activity was sensitive to TLR2 ligands by employing two HEK cell lines expressing either mTLR2 or mTLR4 (see *Materials and Methods*). TLR2 signaling showed a low but significant increase in response to *E. coli*-LPS but not to RAP99-LPS (**Figure 1B**). In contrast, both LPS activated the TLR4 signaling pathway although the *E. coli*-LPS stimulation was stronger (**Figure 1C**). These results demonstrated that RAP99-LPS is a TLR4 ligand.

**Figure 2:**
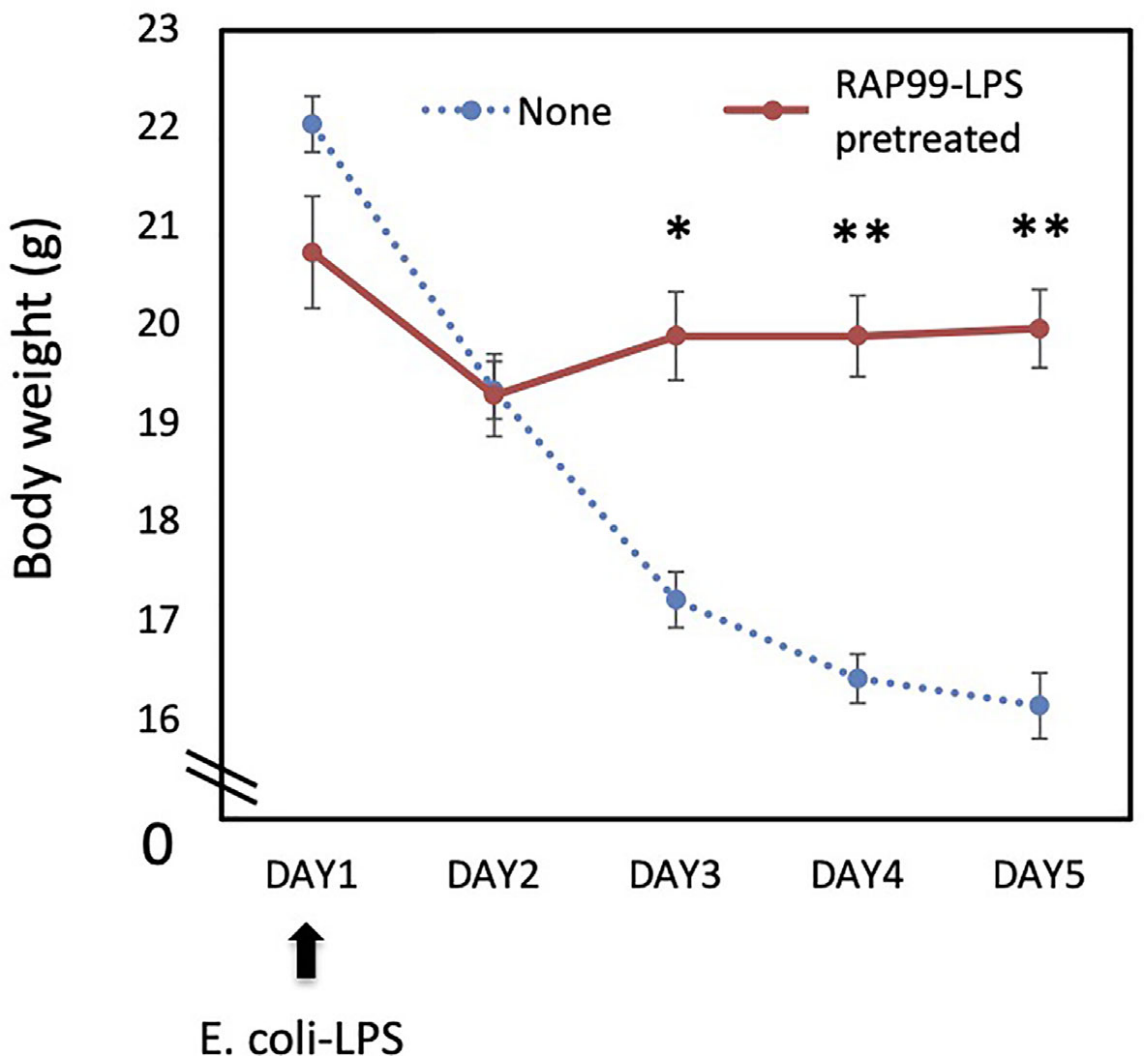
RAP99-LPS pretreatment suppressed *E. coli*-LPS-mediated body weight loss. RAP99-LPS (200 μg/day) or saline was intraperitoneally injected into C57BL/6 mice daily for 5 days followed by the injection of *E. coli*-LPS (200 μg) on day 1 to investigate body weight. Mean scores ± SD are shown. *p < 0.05, **p < 0.01; Student’s t tests. (n = 5 each, Representative data are shown.)

We next investigated whether RAP99-LPS has an antagonist activity to *E. coli*-LPS. We injected RAP99-LPS for 5 days once daily before injecting *E. coli*-LPS (day 1) and then observed the mice for 5 days. Pretreatment with RAP99-LPS significantly suppressed *E. coli*-LPS-mediated weight loss (**Figure 2**), demonstrating the antagonistic activity of RAP99-LPS to *E. coli*-LPS.

### RAP99-LPS Is a TLR4 Agonist That Induces Immune Cell Activation

**Figure 3:**
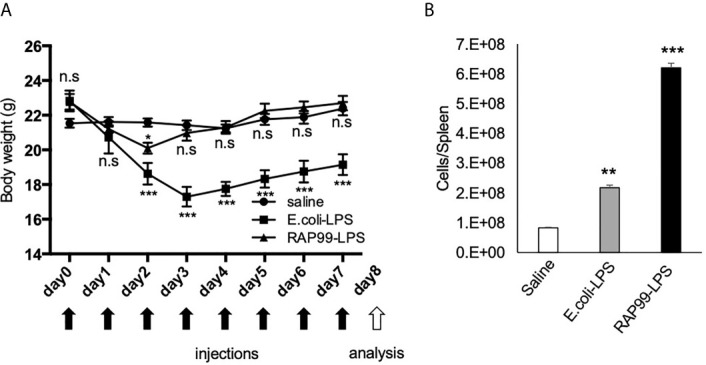
RAP99-LPS treatment induced immune activation. *E. coli*-LPS (200 μg/day), RAP99-LPS (200 μg/day), or saline was intraperitoneally injected into C57BL/6 mice for 8 days to investigate **(A)** body weight and **(B)** the number of splenocytes. Mice injected with *E. coli*-LPS and RAP99-LPS were compared to mice injected with saline. Mean scores ± SD are shown. **p < 0.01; ***p < 0.001; n.s., not significant; Student’s t tests. Saline (n = 4), *E. coli*-LPS (n = 5), RAP-LPS (n = 5). Black arrows: injections. White arrow: analysis. (Representative data are shown.)

Following the *in vitro* results, we investigated whether RAP99-LPS has immune stimulatory activity *in vivo* as a TLR4 agonist. We injected RAP99-LPS and *E. coli*-LPS into mice and investigated body weight and the activation status of immune cells in the spleen. We injected 200 μg RAP99-LPS following a previous study that investigated *E. coli*-LPS (19). We found that RAP99-LPS injections induced transient weight loss, but that the weight recovered later (**Figure 3A**). The weight loss by *E. coli*-LPS injections was higher (**Figure 3A**), but the total number of splenocytes in the spleen was higher after RAP99-LPS injections than after *E. coli*-LPS injections (**Figure 3B**).

RAP99-LPS injections increased DCs (CD11c+), Ly6G/C-CD11b+ cells, Ly6G/C+CD11b+ cells, B cells (CD19+), and NK cells (NK1.1+) more than *E. coli*-LPS injections did. The number of CD4+ T cells (CD44loCD4+ and CD44hiCD4+), CD8+ T cells (CD44loCD8+ and CD44HiCD8+), Treg cells (Foxp3+CD4+), and Ter-119+ cells was also increased more in the spleen where RAP99-LPS was injected (**Supplementary Figure 1**).

**Figure 4:**
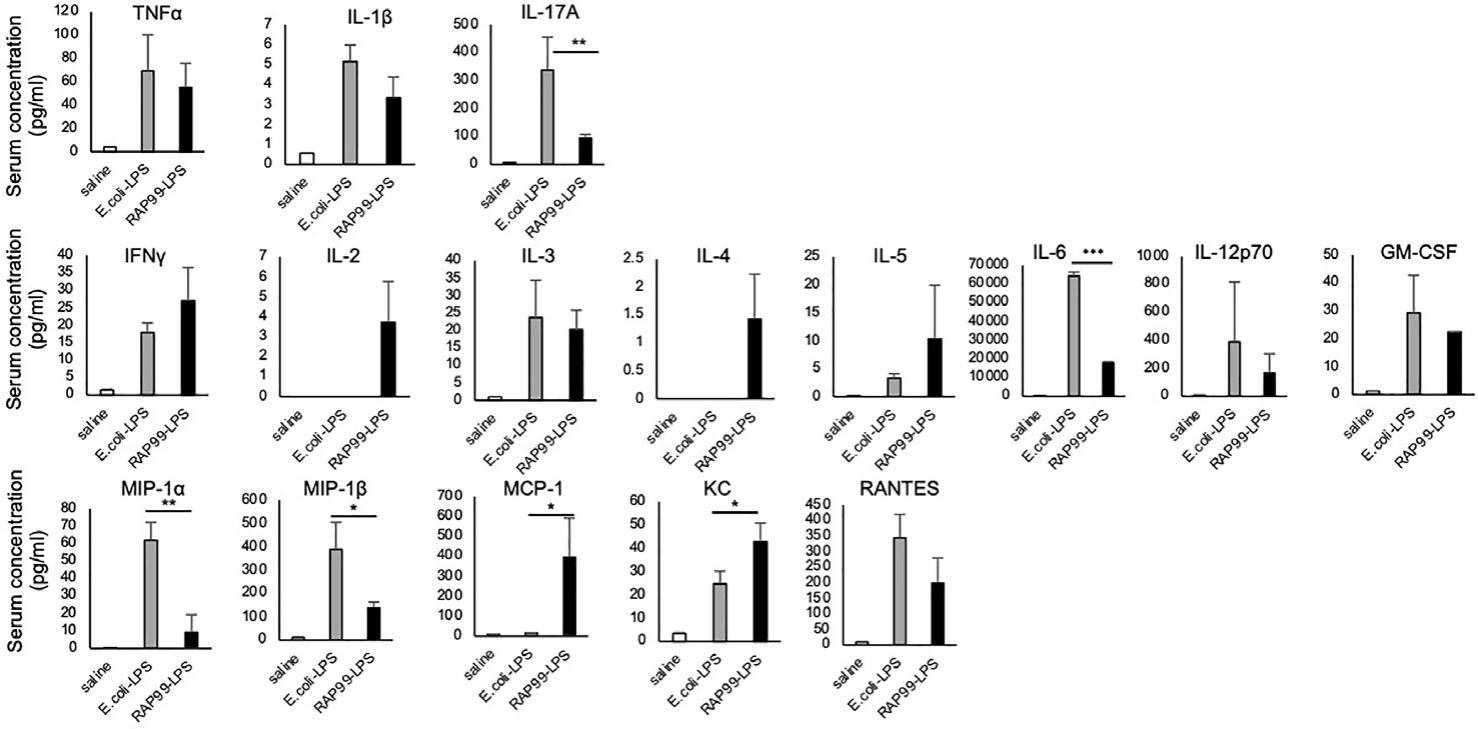
RAP99-LPS induced immune cell activation. *E. coli*-LPS (200 μg/day), RAP99-LPS (200 μg/day), or saline was intraperitoneally injected into C57BL/6 mice followed by an analysis of IL-6 serum levels and serum cytokines and chemokines (pg/ml). Mean scores ± SD are shown. *p < 0.05, **p < 0.01, ***p < 0.001; Student’s t tests. Saline (n = 4), *E. coli*-LPS (n = 5), RAP-LPS (n = 5). (Representative data are shown.)

We also investigated cytokine and chemokine expressions after the RAP99-LPS and *E. coli*-LPS treatments. We found that NF-kB stimulators such as TNFa, IL-1b, and IL-17A; STAT stimulators including IFNg, IL-2, IL-3, IL-4, IL-5, IL-6, IL-12, and GM-CSF; and chemokines such as MIP1a, MIP1b, MCP1, KC, and RANTES were all increased after the treatments, but the levels of IL-6, IL-17A, MIP1a, and MIP1b after RAP99-LPS treatment were less than after *E. coli*-LPS treatment (**Figure 4**).

**Figure 5:**
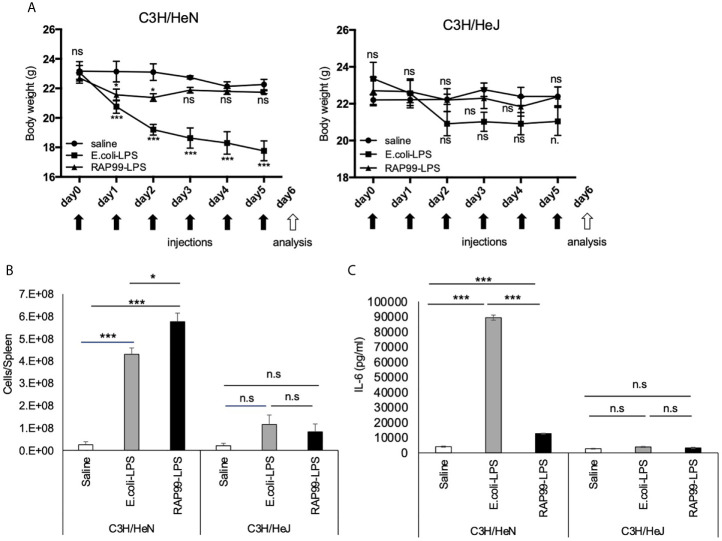
TLR4 is involved in RAP99-LPS-mediated immune activation *in vivo*. *E*. *coli*-LPS (50 μg/day), RAP99-LPS (50 μg/day), or saline was intraperitoneally injected into C3HHe/N mice and C3HHe/J mice for 9 days to investigate **(A)** body weight, **(B)** the number of splenocytes, and **(C)** IL-6 serum level. Mice injected with *E. coli*-LPS and RAP99-LPS were compared with mice injected with saline **(A)**. Mean scores ± SD are shown. Saline (n = 4), *E. coli*-LPS (n = 5), RAP-LPS (n = 5). Black arrows: injections. White arrow: analysis. (Representative data are shown.) *p < 0.05, ***p < 0.005; n.s., not significant.

Because TLR4 is the receptor of LPS, we next investigated whether RAP99-LPS-mediated weight loss and splenomegaly are dependent on TLR4. We employed C3H/HeJ mice for these experiments, because they have no TLR4 signal transduction due to a dominant negative mutation. Consistent with our notion, RAP99-LPS injections induced only negligible transient splenomegaly in C3H/HeJ mice, unlike in control C3H/HeN mice (**Figures 5A, B** **)**. We also showed that IL-6 was hardly increased in C3H/HeJ mice even after six injections of RAP99-LPS (**Figure 5C**). Thus, treatment with RAP99-LPS, a TLR4 stimulator, induced immune activation *via* TLR4.

### RAP99-LPS Treatment Suppressed Lung Metastasis of B16F1 Cells

**Figure 6:**
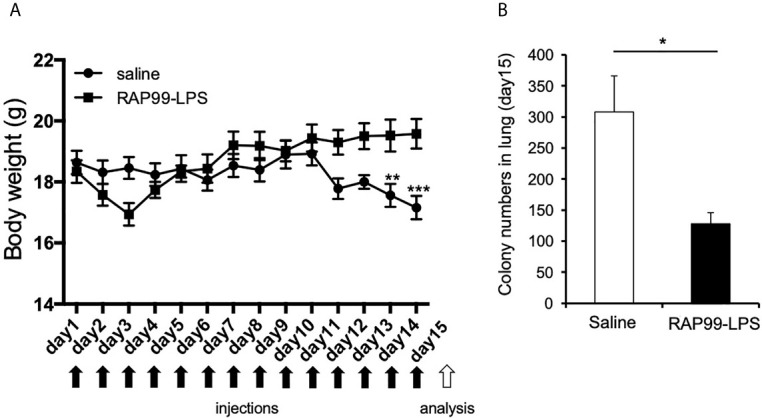
RAP99-LPS treatment suppressed body weight loss and lung metastasis of B16F1 cells. 1 × 10^6^ B16F1 cells were intravenously injected into C57BL/6 mice with or without RAP99-LPS (200 μg/day) injections for 14 days. **(A)** Body weight. **(B)** The mice were sacrificed, and B16F1 cell colonies in the lungs were measured. Mean scores ± SD are shown. *p < 0.05, **p < 0.01, ***p < 0.001; Student’s t tests. Black arrows: injections. White arrow: analysis. (n = 4 each; Representative data are shown.)

We next investigated the beneficial effects of RAP99-LPS *in vivo*. We investigated whether the RAP99-LPS-induced immune activation suppresses lung metastasis of B16F1 cells, because cytokines, macrophages, NK cells, and gdT cells are involved in the immune response against these cells (20–23). B16F1 cells were intravenously injected into mice, followed by injections of RAP99-LPS. RAP99-LPS treatment significantly suppressed body weight loss and lung metastasis of B16F1 cells (**Figure 6**). Consistently, RAP99-LPS treatment significantly increased spleen weight even in mice with B16F1 cells (data not shown). Therefore, RAP99-LPS enhances anti-tumor responses *in vivo via* TLR4.

### RAP99-LPS Pretreatment Enhanced the Expression of TLR3-Mediated Chemokines but Suppressed the Expression of NF-kB Stimulators and STAT3 Stimulators

**Figure 7:**
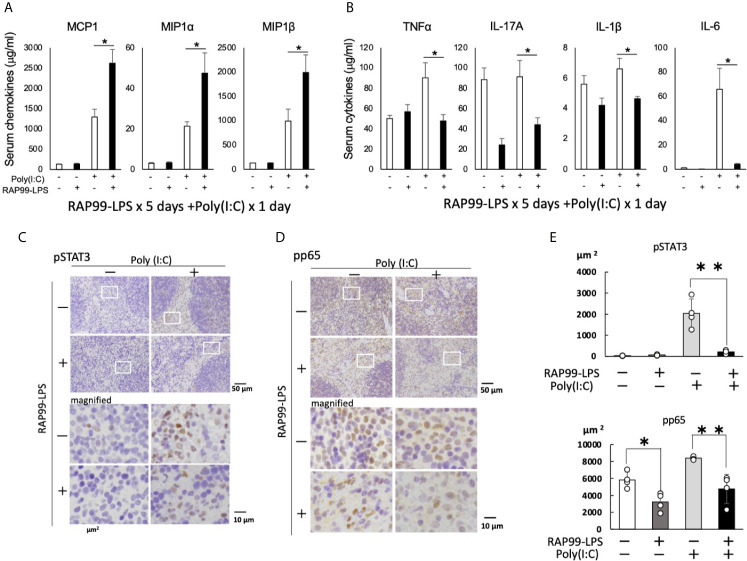
RAP99-LPS pretreatment enhanced the expression of chemokines and suppressed the expression of NF-kB- and STAT3-stimulating cytokines after TLR3 ligand injection. RAP99-LPS (200 μg/day) or saline was intraperitoneally injected into C57BL/6 mice every day 5 times followed by the injection of poly(I:C) (200 μg) on day 6. Serum chemokine **(A)** and cytokine **(B)** levels were investigated 6 h later. Mean scores ± SD are shown. *p < 0.05; Student’s t tests. Immunohistochemistry of spleens was performed with anti-phospho-STAT3 antibody **(C)** and anti-phospho-NF-kB antibody **(D)**. **(E)** Quantified data of **(C)** and **(D)**. The area ratio of nuclei with pStat3 or pp65 (DAB + area) is presented per 10,000μm^2^ (Hematoxylin + area). (n = 4 each; Representative data are shown.) *p < 0.05, **p < 0.01.

We finally investigated whether pretreatment with RAP99-LPS enhances poly(I:C)-mediated immune responses, because we hypothesized that RAP99-LPS enhances anti-viral responses against RNA viruses such as SARS-CoV2. The pretreatment of RAP99-LPS significantly enhanced the expression of several chemokines including MCP1, MIP1a, and MIP1b, while suppressing the expression of NF-kB stimulators and STAT3 stimulators such as TNFa, IL-1b, IL-17A, and IL-6, which play a role in cytokine storms *via* activation of the IL-6 amplifier (**Figures 7A, B** **)**. The IL-6 amplifier enhanced NF-kB activation machinery in non-immune cells stimulated by the simultaneous activation of STAT3 and NF-kB pathways (24). Immunohistochemistry of the spleens showed that the phosphorylation of STAT3 and NF-kB was correlated with the expression of NF-kB stimulators and STAT3 stimulators after stimulation with RAP99-LPS and/or poly (I:C) (**Figures 7C–E**). These results suggest that RAP99-LPS pretreatment might enhance the local accumulation of effector immune cells by enhancing TLR3-mediated chemokines after RNA virus infection and suppresses cytokine storm induction by reducing the level of NF-kB stimulators and STAT3 stimulators, which otherwise damages organ homeostasis.

## Discussion

We here showed that unlike other *Rhodobacter* LPS, RAP99-LPS, the LPS of *Rhodobacter azotoformans* BP0899, is a TLR4 agonist that activates immune cells and secretes chemokines and cytokines *in vivo* and *in vitro*. RAP99-LPS showed some beneficial effects *in vivo* including (i) the suppression of weight loss induced by *E. coli*-LPS endotoxin shock, (ii) the inhibition of B16F1 cell lung metastasis, and (iii) the induction of TLR3-mediated chemokines and the suppression of NF-kB stimulators and STAT3 stimulators involved in cytokine storms. Because the extract of *Rhodobacter azotoformans* is used as a health supplement for humans and animals in Japan to regulate immune responses to suppress diseases, our results could be a proof of concept of this supplement *in vivo*. It should be noted, however, that the doses used in this study are much higher than those of off-the-counter supplements used by humans and animals.

We showed that RAP99-LPS stimulates the TLR4 but not TLR2 signaling pathway *in vitro*. Consistently, RAP99-LPS activated immune cells *via* TLR4 like *E. coli*-LPS, because we found RAP99-LPS-mediated weight loss and splenomegaly were significantly suppressed in C3H/HeJ mice, which lack TLR4-mediated signal transduction, but not in control C3H/HeN mice. We also showed that the structure of RAP99-LPS is similar to that of *E. coli*-LPS, but it also contains a novel lipooligosaccharide consisting of a shorter glycan structure containing glucuronic acid instead of heptose (13). Indeed, RAP99-LPS treatment *in vivo* activated various immune cells in the innate and adaptive immune systems including DCs, macrophages, neutrophils, NK cells, gdT cells, B cells, and T cells. The RAP99-LPS activity for immune activation was three times weaker than that of *E. coli*-LPS based on serum IL-6 concentrations, but twice as strong based on splenomegaly (**Figures 1** and **2**). According to RIKEN Reference Database of Immune Cells (http://refdic.rcai.riken.jp/welcome.cgi), TLR4 is mainly expressed on epithelial cells, fibroblasts, stromal cells, B cells, dendritic cells, macrophages, and mast cells, but is also expressed lowly on T cells, NK cells, and NKT cells. Therefore, we hypothesize that RAP99-LPS has both direct and indirect effects on immune cells, including CD4+T cells, CD8+T cells, T reg cells, gdT cells, and NK cells, with at least one of the indirect effects mediated by cytokines.

We also found high concentrations of chemokines such as MCP1 and KC after RAP99-LPS treatment compared with *E. coli*-LPS treatment (**Figure 2C**). These results strongly suggest that immune cells are differentially activated by RAP99-LPS and *E. coli*-LPS treatment even though both LPS are TLR4 ligands. Despite both being TLR4 ligands and having similar purity, we found only *E. coli*-LPS transduced weak TLR2 signaling *in vitro* (**Figure 1B**), suggesting different downstream signaling. Moreover, we found *E. coli*-LPS transduced more TLR4 signaling compared with RAP99-LPS *in vitro* (**Figure 1C**), suggesting different TLR4 agonist activities. We hypothesize that these differences explain the different cytokine induction patterns between *E. coli*-LPS and RAP99-LPS.

The extract of *Rhodobacter azotoformans* is sometimes used as a health supplement. In the present study, we showed three health benefits of RAP99-LPS in mouse models. *E. coli*-LPS injection causes an endotoxin shock that induces a cytokine storm, weight loss and sometimes leads to death (25). However, like other LPS isolated from *Rhodobacter* (10–12), mice pretreated with RAP99-LPS did not show weight loss after *E. coli*-LPS injection. This finding suggests that RAP99-LPS supplementation can prevent the food poisoning induced by bacteria that activate TLR4, including *E. coli* O157. Both RAP99-LPS and *E. coli*-LPS bind to TLR4, indicating the molecular mechanism of the RAP99-LPS-mediated weight loss suppression involves TLR4-receptor competition (26). Moreover, because more TLR4 signaling induces more endotoxin tolerance (27) and because we found *E. coli*-LPS transduced more TLR4 signaling compared with RAP99-LPS, we hypothesize that not only TLR4-receptor competition but also LPS tolerance or other factors are involved in the preventative effects of RAP99-LPS against *E. coli*-LPS as well as TLR3 ligands. Indeed, we found no obvious antagonistic effect, at least in HEK-Blue TLR4 cells (**Supplementary Figure 5**).

Because resistance to the metastasis of tumor cells is dependent on cytokines, macrophages, NK cells, and gdT cells (20–23), we investigated the anti-tumor activity of RAP99-LPS using a B16F1 lung metastasis model and found that RAP99-LPS treatment significantly suppressed the metastasis, though we did not show if this suppressive effect is mediated by TLR4. Moreover, pretreatment with RAP99-LPS increased the expression of several chemokines, including MCP1, MIP1a, and MIP1b, but suppressed the expression of NF-kB and STAT3 stimulators, including IL-1b, TNFa, IL-17A, and IL-6, after the injection of a TLR3 ligand, poly(I:C). Because poly(I:C) injection mimics RNA viral infections *in vivo*, these results suggested that people taking *Rhodobacter azotoformans* extract, including RAP99-LPS, daily might have activated immune systems against RNA viruses. Interestingly, excessive concentrations of NF-kB and STAT3 stimulators induce the lethal cytokine storm seen in COVID-19 patients (24). Although “cytokine storm” is a loosely defined term, we recently defined it as a poor prognosis induced by inflammation (24). We hypothesize that the body weight loss alone seen in this model is not a cytokine storm, even though the weight loss is induced by excessive cytokine production after TLR4 stimulation. Overall, pretreatment with RAP99-LPS might have beneficial effects during viral infections including SARS-CoV2.

In conclusion, we demonstrated that RAP99-LPS, which is a component in the extract of *Rhodobacter azotoformans*, activates immune cells as a TLR4 agonist. This is the first report describing LPS of *Rhodobacter* as a TLR4 agonist. Importantly, we found that treatment with RAP99-LPS inhibited B16F1 tumor cell metastasis in the lung and enhanced the expression of poly(I:C)-mediated chemokines but suppressed the expression of cytokines involved in lethal cytokine storms. These results suggest that the extract of *Rhodobacter azotoformans*, which is used as a health supplement in Japan, might have beneficial effects on tumor lung metastasis as well as on virus infections including SARS-CoV2.

## Data Availability Statement

The datasets presented in this study can be found in online repositories. The names of the repository/repositories and accession number(s) can be found in the article/**Supplementary Material**.

## Ethics Statement

The animal study was reviewed and approved by Hokkaido University Institute for Genetic Medicine. Written informed consent was obtained from the owners for the participation of their animals in this study.

## Author Contributions

Experiments: KM, DK, RH, MU, NA, RY, J-JJ, YTa, and MM. Checking: KM, YH, YN, SF, YTo, NT, HT, SA, YTa, and MM. All authors contributed to the article and approved the submitted version.

## Funding

This work was supported by JSPS KAKENHI (DK and MM), AMED (MM), the Joint Usage/Research Center Institute for Genetic Medicine, Hokkaido University (MM), Glaxo Smith Kline Foundation (YTa), Kowa Life Science Foundation (YTa), and Takeda Science Foundation (MM), and partly supported by the Photo-excitonix Project and the project of young researcher promotion in Hokkaido University (MM).

## Conflict of Interest

YH, YN, SF, YT and NT were employed by TFK Co., Ltd.

The remaining authors declare that the research was conducted in the absence of any commercial or financial relationships that could be construed as a potential conflict of interest.

## Supplementary Material

Supplementary Figure 1 Flowcytometry of spleen cells was performed on mice treated with *E. coli*-LPS (200 μg/day), RAP99-LPS (200 μg/day), or saline. Mean scores ± SD are shown. *p < 0.05, **p < 0.01, ***p < 0.001; Student’s t tests. Saline (n = 4), *E. coli*-LPS (n = 5), RAP-LPS (n = 5). (Representative data are shown.)

Supplementary Figure 2 The gating strategy of T cells for the flow cytometer analysis. (Representative data are shown.)

Supplementary Figure 3 The gating strategy of myeloid cells for the flow cytometer analysis. (Representative data are shown.)

Supplementary Figure 4 The gating strategy of Treg cells for the flow cytometer analysis. (Representative data are shown.)

Supplementary Figure 5 The gating strategy of NK cells and Ter119 for the flow cytometer analysis. (Representative data are shown.)

Supplementary Figure 6 RAP99-LPS does not function as an antagonist of *E. coli*-LPS. OD630 was analyzed 6 h after treating HEK-Blue™ mTLR4 cells with *E. coli*-LPS or RAP99-LPS. Mean scores ± SD are shown. ***p < 0.005, Student’s t test. (n = 5 each; Representative data are shown.)
