# Supplementary figures and images for: Rhodobacter azotoformans LPS (RAP99-LPS) Is a TLR4 Agonist That Inhibits Lung Metastasis and Enhances TLR3-Mediated Chemokine Expression
Source: Front Immunol. 2021 May 25;12:675909. doi: 10.3389/fimmu.2021.675909 (PMC8185171; doi:10.3389/fimmu.2021.675909)

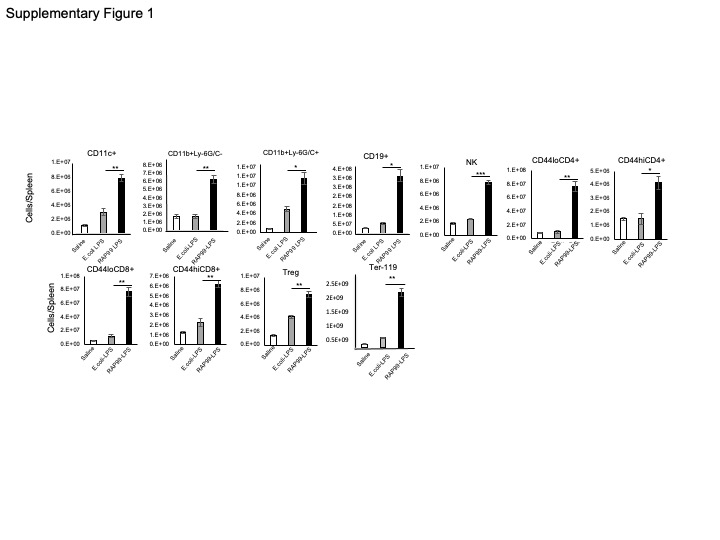

Supplement: Supplementary Figure 1 — Flowcytometry of spleen cells was performed on mice treated with E. coli-LPS (200 μg/day), RAP99-LPS (200 μg/day), or saline. Mean scores ± SD are shown. *p < 0.05, **p < 0.01, ***p < 0.001; Student’s t tests. Saline (n = 4), E. coli-LPS (n = 5), RAP-LPS (n = 5). (Representative data are shown.) [file Image_1.jpeg]

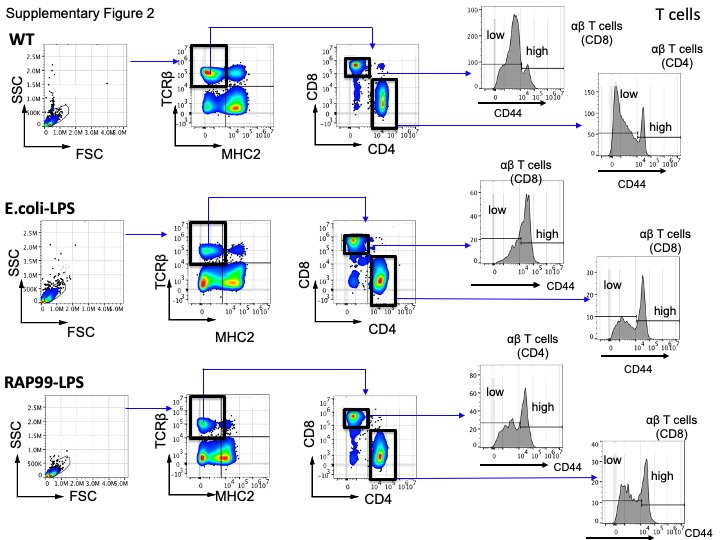

Supplement: Supplementary Figure 2 — The gating strategy of T cells for the flow cytometer analysis. (Representative data are shown.) [file Image_2.jpeg]

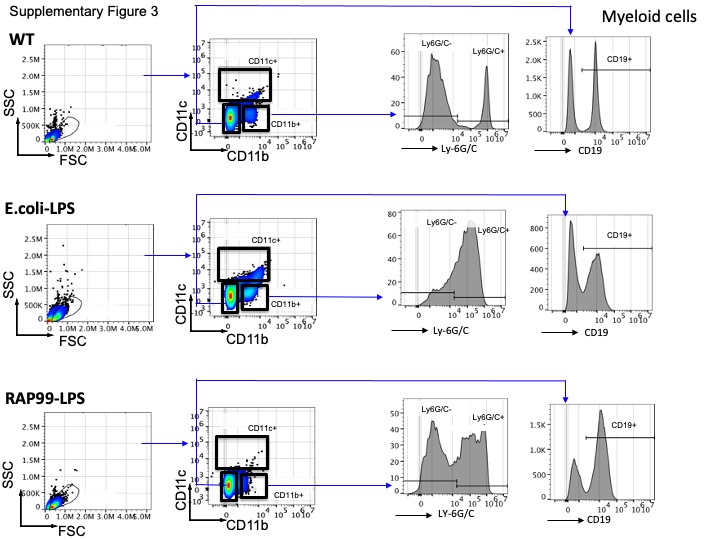

Supplement: Supplementary Figure 3 — The gating strategy of myeloid cells for the flow cytometer analysis. (Representative data are shown.) [file Image_3.jpeg]

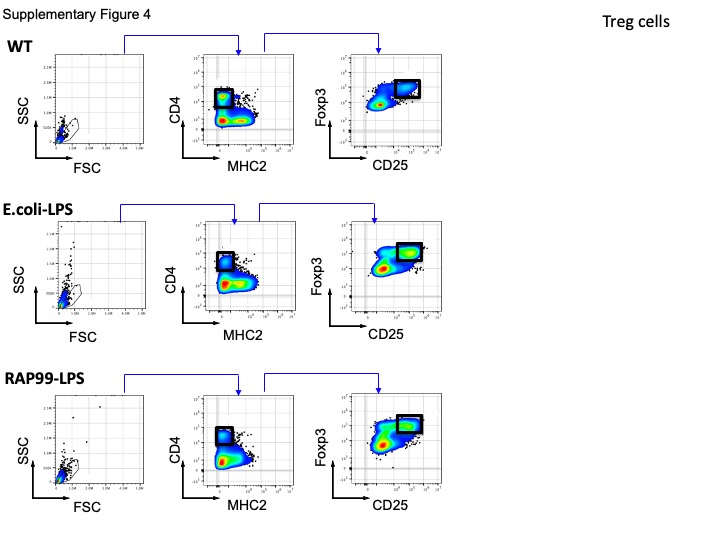

Supplement: Supplementary Figure 4 — The gating strategy of Treg cells for the flow cytometer analysis. (Representative data are shown.) [file Image_4.jpeg]

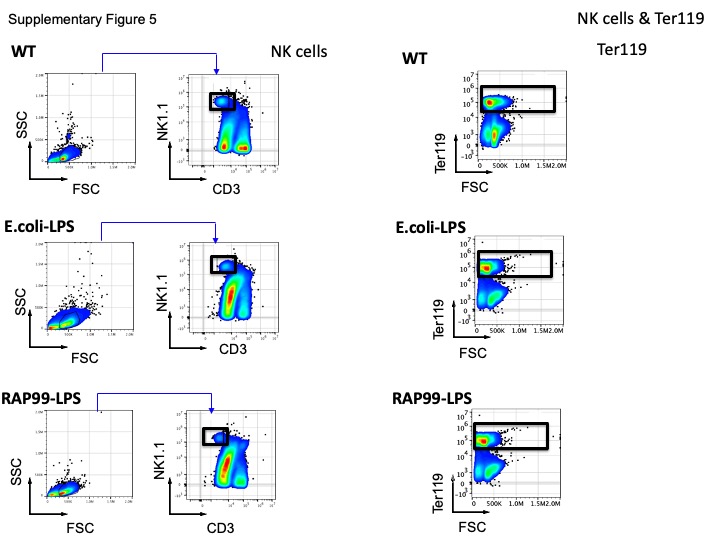

Supplement: Supplementary Figure 5 — The gating strategy of NK cells and Ter119 for the flow cytometer analysis. (Representative data are shown.) [file Image_5.jpeg]

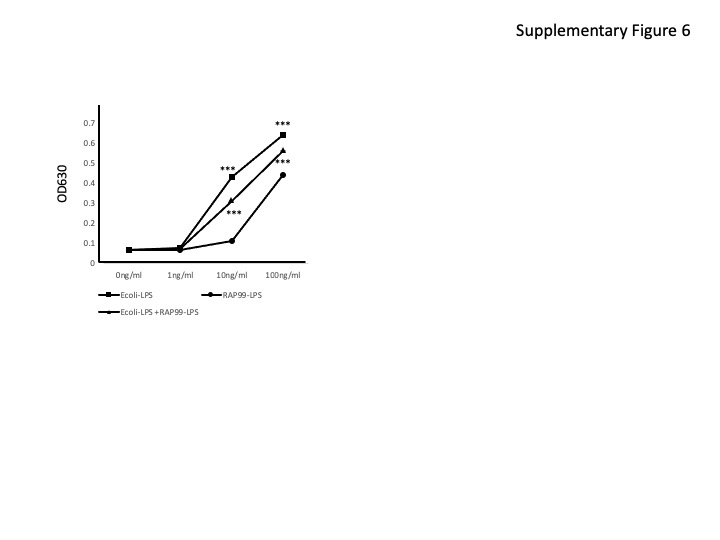

Supplement: Supplementary Figure 6 — RAP99-LPS does not function as an antagonist of E. coli-LPS. OD630 was analyzed 6 h after treating HEK-Blue™ mTLR4 cells with E. coli-LPS or RAP99-LPS. Mean scores ± SD are shown. ***p < 0.005, Student’s t test. (n = 5 each; Representative data are shown.) [file Image_6.jpeg]
